# Influential factors affecting nursing performance amid COVID-19: A cross-sectional study on nurse preparedness for infectious diseases

**DOI:** 10.1016/j.ijnsa.2024.100239

**Published:** 2024-09-06

**Authors:** Kyung-sook Cha, Dohyun Lee

**Affiliations:** a Department of Nursing, Sun Moon University, South Korea; b Department of Kinesiology and Recreation Administration, North Carolina Central University, PO Box 19542, Durham, NC 27707, USA

**Keywords:** Attitude, COVID-19, Infectious disease, Nursing, Self-efficacy, Structural equation modeling

## Abstract

**Background:**

The emergence of infectious diseases such as SARS, MERS, and COVID-19 underscores the need for effective nursing preparedness.

**Objectives:**

This cross-sectional study sought to pinpoint the factors that impact nursing performance during the COVID-19 pandemic, focusing on nurses' self-efficacy, work environment, knowledge, and attitudes.

**Design:**

The study utilized a cross-sectional design.

**Settings & participants:**

Between December 13, 2021, and January 21, 2022, an online and offline survey was conducted with 314 nurses who provided in-person care in tertiary and general hospitals throughout South Korea.

**Methods:**

Using *t*-tests and ANOVA, the research compared nursing performance based on various demographic and work-related characteristics like age, gender, education, marital status, and other working conditions. A research model was formulated via structural equation modeling, positioning nursing performance as the dependent variable. The independent variables included career experience (indirect effect), work environment (indirect effect), COVID-19 knowledge (indirect effect), attitude toward COVID-19 (direct effect), and self-efficacy (direct effect). Data analysis was carried out using SPSS 26 and AMOS 28.

**Results:**

The study demonstrated that self-efficacy and attitude toward COVID-19 significantly influence nursing performance, as reflected by robust critical ratios (CR) for self-efficacy (CR = 11.291, *p* < 0.001) and attitude (CR = 5.133, *p <* 0.001). They account for 43 % (*R*^2^ = 0.43) of the variability of nursing performance. Self-efficacy was positively predicted by clinical experience (CR = 3.160, p = 0.002) and work environment (CR = 4.328, *p <* 0.001), while attitude was similarly influenced (CR = 3.557 and 2.926, respectively). However, clinical experience and work environment only explained 8 % (*R*^2^ = 0.08) of self-efficacy and 16 % (*R*^2^ = 0.16) of attitude. Knowledge about COVID-19 exhibited a statistically insignificant influence in the dynamics.

**Conclusions:**

This study, conducted among Korean nurses during the COVID-19 pandemic, reveals that self-efficacy and attitudes towards COVID-19 are key predictors of nursing performance, overshadowing knowledge's influence. These findings suggest the critical role of psychological factors in healthcare delivery during crises and underscore the need for enhanced focus on developing self-efficacy and positive attitudes in nursing education and professional development. Additionally, demographic and professional variables, including age, clinical experience, educational level, and marital status affect nursing performance.

**What is already known about the topic**

•Self-efficacy significantly impacts nursing performance in high-stress settings.

•Work environment and clinical experience influence nursing performance through self-efficacy and attitudes.

•Prior studies have seldom explored the combined effects of psychological and environmental factors during a pandemic.

Alt-text: Unlabelled box

**What this paper adds**

•This study demonstrates that self-efficacy and attitudes towards COVID-19 are the most significant predictors of nursing performance, collectively explaining 43 % of the variance.

•The findings reveal that knowledge about COVID-19, while essential, does not significantly predict nursing performance, highlighting the greater importance of psychological factors during crises.

•The research underscores the need for nursing education programs to prioritize the development of self-efficacy and positive attitudes to enhance performance during future health emergencies.

Alt-text: Unlabelled box

## 1 Introduction

Since the early 21st century, infectious diseases such as severe acute respiratory syndrome (SARS), Middle East Respiratory Syndrome (MERS), and Coronavirus disease 2019 (COVID-19) have significantly impacted public health and medical research. In early outbreaks, medical personnel struggled due to the lack of established countermeasures, with nurses facing substantial pressure to make swift, informed decisions about new symptoms (Xu et al., 2021).

COVID-19, identified in December 2019, rapidly became a global pandemic. South Korea experienced a significant outbreak in Daegu linked to a religious event but managed it effectively through extensive testing, tracing, and quarantine measures, avoiding complete lockdowns. This response highlighted a strategic shift towards endemic management by early 2022.

COVID-19 primarily spreads through respiratory droplets, infecting through the respiratory system, eyes, nose, and mouth, especially after contact with contaminated surfaces. Medical professionals are at higher risk due to close contact with infected individuals (Korea Disease Control and Prevention Agency, 2021). These challenges are global, affecting nurses everywhere (Danesh et al., 2021). Nurses adapted to changing protocols, often working without knowing patients' COVID-19 status due to initial testing shortages and personal protective equipment (PPE) shortages, necessitating improvised measures (Ding et al., 2022). As a result, it is imperative for front-line nurses to employ enhanced preventive measures, including the use of PPE (Danesh et al., 2021; Korea Disease Control and Prevention Agency, 2021). Once adequately protected, the emphasis shifts to ensuring the effective delivery of care. Given the pivotal role of nurses in this context, understanding the determinants of their performance becomes crucial, particularly in readiness for potential future outbreaks of unknown infectious diseases.

The primary role of nursing entails identifying patient needs using professional expertise, and confidently delivering the necessary care to promote well-being and facilitate healing. In this context, nursing performance encompasses a myriad of factors including understanding patients, clinical judgment, coping mechanisms, professional development, people orientation, resource management, ethical considerations, collaboration, self-confidence, self-regulation, adaptability, influence, and the capacity to nurture others (Han, 2007; Nabizadeh-Gharghozar et al., 2021; Schrimmer et al., 2019). Building on the previous discussion, Lee (2001) explores the concept of nursing performance by highlighting the importance of effectively applying knowledge across similar situations. This involves not only the ability to simplify complex relationships but also to identify connections among seemingly unrelated pieces of information. Therefore, nursing performance is fundamentally a blend of knowledge, skills, and attitudes that nurses bring together to provide quality care in clinical settings. This comprehensive approach underscores the essence of nursing outcomes, which are achieved through the integration of these critical components within the healthcare environment. Enhanced nursing performance invariably leads to improved patient comfort and reduces the potential for complications. It is therefore imperative to prioritize and enhance the performance metrics within the nursing profession.

Past research suggests that both personal characteristics and the environmental attributes of hospitals play a significant role in shaping nursing performance (Song et al., 2006; Jo and Sung, 2018). On the personal front, factors such as knowledge of disease, self-concept, and resilience have been identified as key determinants (Alonazi et al., 2023; Farhadi et al., 2021; Kim and Chang, 2022; Miao et al., 2024; Varshney and Varshney, 2024). Environmentally, leadership and the overall hospital working environment emerge as influential elements in determining nursing performance (Balsanelli et al., 2018; Germain and Cummings, 2010; Silva et al., 2017).

During the pandemic, medical personnel were often tasked with treating individuals exhibiting severe COVID-19 symptoms immediately upon presentation, sometimes even at the nearest available bedside. These healthcare workers frequently found themselves in precarious situations, operating without clear knowledge of the disease's transmission routes and in the absence of a definitive cure. Given that COVID-19 is highly infectious, nurses were mandated to don level D protective gear and attend to patients in negative pressure isolation rooms. Despite these challenging conditions, auxiliary support for these frontline workers was notably limited while they delivered medical care and assisted with daily living activities (Korea Disease Control and Prevention Agency, 2021; Park et al., 2021). It is crucial to identify the factors that significantly impact nursing performance under these trying circumstances.

When confronted with emergent infectious diseases like COVID-19, nurses face the imperative to quickly assimilate new knowledge about the ailment. This understanding not only facilitates rapid and accurate decision-making but also bolsters their confidence in both self-protection and patient care. Therefore, a nurse's ability to acquire and integrate knowledge about novel diseases plays a crucial role in shaping their overall nursing performance.

Self-efficacy refers to an individual's confidence in their ability to execute tasks. Those with high levels of self-efficacy are more likely to take ownership of their work and see it through to completion (Chang, 2015). In the nursing context, self-efficacy refers to a nurse's confidence in their ability to deliver effective care. Previous studies have highlighted the crucial role of nurses' self-efficacy in managing patient care, especially during the increased workloads and stressful conditions of the COVID-19 pandemic (Lim et al., 2022; Pragholapati, 2020; Yang et al., 2022). This confidence not only enhances nurses' performance but also fosters patient trust, which in turn leads to greater acceptance and active engagement in the provided care (Sharour et al., 2022). Consequently, it is imperative for nurses to possess a high degree of self-efficacy to both cultivate patient trust and promote optimal cooperation with care regimens.

The quality of the nursing work environment is intrinsically linked to nurses' performance (Sung and Lee, 2017). This environment encompasses factors such as working hours, allotted break times, patient-to-nurse ratios, and the support provided by the hospital. A superior work environment, complemented by robust hospital support, enables nurses to execute their duties with greater precision and efficiency.

This study seeks to establish foundational data to inform nursing education and training in the context of emerging infectious diseases, using the backdrop of COVID-19. Specifically, the focus is on discerning the factors that impact the nursing performance of those tending to individuals infected with COVID-19. The research aims to explore the relationships between nursing performance and variables such as nurses' self-efficacy, the nursing work environment, knowledge about COVID-19, and attitudes toward COVID-19. The overarching objectives of the study are outlined below:

First, the study assesses disparities in nursing performance based on the general attributes of Korean nurses during the COVID-19 pandemic.
Second, the research gauges the levels of nurses' self-efficacy, nursing work environment, knowledge about COVID-19, attitude toward COVID-19, and overall nursing performance among Korean nurses during the pandemic.
Third, the study investigates the paths and magnitudes of associations among nurses' self-efficacy, nursing work environment, knowledge about COVID-19, attitudes toward COVID-19, and nursing performance in Korea.
Fourth, the research identifies the most influential variables that affect nursing performance during the COVID-19 outbreak in Korea.

Completing the study objects, the study uniquely examines how self-efficacy, work environment, knowledge, and attitudes collectively influence nursing performance during the COVID-19 pandemic. Previous studies often focus solely on either psychological or environmental factors, but rarely consider their combined effects in the context of a pandemic.

## 2 Methods

### 2.1 Study design and participants

This study adopted a cross-sectional design, deploying both online and offline surveys. The target participants were nurses from tertiary and general hospitals across Korea, who were actively involved in patient care.

The sample size was meticulously determined based on the requirements for a *t*-test and structural equation modeling. For the *t*-test, a power analysis indicated a need for at least 176 participants given a significance level of 0.05, an effect size of 0.5, and a power of 0.95. Regarding structural equation modeling, Yu (2012) recommended a participant range of 200–400 when employing the Maximum Likelihood [ML] method. Other literature further supports a baseline of 100 subjects for structural equation modeling (Anderson and Gerbing, 1988; Ding et al., 1995; Tabachnick et al., 2001).

Aiming to ensure robust statistical analysis and account for potential dropouts, the study sought to enlist 350 participants. By employing both online and offline survey methods, we hoped to facilitate participation without geographical limitations. From this effort, 214 nurses participated through the online platform, while 151 opted for the offline method, culminating in 365 total respondents. However, upon excluding 51 participants due to incomplete responses, 314 participants were finalized for analysis.

### 2.2 Measurements

#### 2.2.1 General characteristics

The general characteristics of the nurses in this study encompass a variety of factors. Personal and professional characteristics include age, clinical experience, gender, education, marital status, the type of hospital where the nurse is employed, position, affiliated department, and work shifts. COVID-19 related characteristics are the presence of a nationally designated isolation bed in the nurse's hospital, training received on emerging infectious diseases, education on the proper use of personal protective equipment specifically for COVID-19, and hands-on nursing experience with people with the illness. These variables were crucial for understanding the demographic and professional background of the participants, allowing for a detailed analysis of how these factors relate to nursing performance.

#### 2.2.2 Nursing performance

Nursing performance was assessed using a tool adapted by Choi (2005) from a nursing performance instrument originally crafted by Lee et al. (1990), which itself was grounded in Schwirian's (1978) Six-Dimension Scale. This instrument is comprehensive, containing 45 items distributed across several domains: 11 items focus on the nursing process, another 11 on nursing skills, eight delve into education and cooperation, six address interpersonal relationships and communication, and the remaining nine pertain to professional development. Respondents rate each item on a five-point Likert scale, where a score of five signifies "very good" and a score of one denotes "very bad." Consequently, a higher average score indicates superior nursing performance. It's noteworthy that the instrument demonstrated strong reliability, with a Cronbach's α of 0.92 in Choi's (2005) study and an even more robust Cronbach's α of 0.97 in the current research.

#### 2.2.3 Self-efficacy

The study employed a research instrument that Jung (2007) adapted from the self-efficacy measurement scale designed by Sherer et al. (1982). This instrument comprises 17 items scored on a five-point scale, where ``always'' equates to five points and ``not at all'' corresponds to one point. Higher scores indicate increased self-efficacy. In Jung's (2007) study, the reliability coefficient, Cronbach's α, was 0.94; similarly, in the present study, Cronbach's α was 0.94.

#### 2.2.4 Nursing work environment

The study utilized the Korean version of the Practice Environment Scale of Nursing Work Index (K-PES-NWI) as developed and validated by Cho et al. (2011) in Korea. This adaptation is based on Lake's (2002) Practice Environment Scale of Nursing Work Index (PES-NWI). The scale encompasses 29 items. Specifically, it includes nine items addressing nurses' involvement in hospital administration, nine items focusing on the foundation for quality nursing, four items related to the capability, leadership, and support nurses receive from nursing managers, four items assessing the adequacy of workforce and material resources, and three items evaluating the cooperative relationships between nurses and doctors. Responses are captured on a Likert scale ranging from one (strongly disagree) to four (strongly agree), with higher scores signifying a more favorable evaluation of the nursing work environment. Importantly, Cho et al. (2011) reported a Cronbach's α value of 0.93 for this scale, a reliability metric mirrored in the current study (Cronbach's α = 0.93).

#### 2.2.5 Knowledge related to COVID-19

The research utilized an instrument adapted and enhanced by Kim (2021). This adaptation was based on the COVID-19 Response Guidelines from the Korea Disease Control and Prevention Agency (2021). The tool is comprised of 20 items, structured as follows: four items related to the cause and incubation period, four focusing on the transmission route and judgment criteria, four concerning symptoms and treatment, and eight pertaining to isolation and release procedures. Participants are directed to respond to each item with either `yes' or `no'. Correct answers are attributed one point, while incorrect ones are given zero. Thus, a higher composite score signifies a deeper knowledge of the topic. The instrument's reliability in this context was validated with a Cronbach's α of 0.71.

#### 2.2.6 Attitudes related to COVID-19

The study employed an instrument originally designed by Park (2006) to measure attitudes towards avian influenza. This tool was subsequently adapted by Baik (2020) to gauge attitudes towards COVID-19. Comprising 12 items, the instrument utilizes a five-point Likert scale, with responses spanning from one point for 'strongly disagree' to five points for 'strongly agree'. Higher scores indicate a more optimistic attitude towards effective prevention and management of COVID-19 infections. While Baik's (2020) research reported a reliability coefficient of Cronbach's α = 0.94 for the instrument, the current study achieved a Cronbach's α value of 0.75.

### 2.3 Data collection

Data collection took place from December 13, 2021, to January 21, 2022, amidst the COVID-19 pandemic. For the online survey component, an announcement seeking participants was shared on a social network bulletin frequented by a substantial number of nurses in Korea. Interested nurses, upon consenting to partake, were provided a link to voluntarily complete the questionnaire.

In the offline phase, permissions were obtained from the nursing departments of tertiary and general hospitals in Korea after a thorough briefing on the study's objectives, methodology, and data collection procedures. Hospital-based nurses were then informed of the study's aim and methodology. Questionnaires were distributed to those who expressed willingness to participate. All participants were assured of the anonymity of their responses and informed that the collected data would serve exclusively for research objectives. Furthermore, they were apprised of their right to discontinue participation at any juncture.

Consent forms were completed by participants, followed by the self-administration of the questionnaires. After processing, valid responses from 314 participants were earmarked for analysis. Importantly, this research obtained the requisite approval from the Institutional Review Board associated with the first author's affiliated university (SM-202111-070-1).

### 2.4 Data analysis

There was no missing data. The study computed the mean and standard deviation for both participants' general attributes and measurement variables. To discern differences in nursing performance based on general characteristics, *t*-tests and ANOVA were employed (Table 1). For post hoc comparisons in ANOVA, Sheffe's method was utilized. The internal consistency of the measurement scales was ascertained using Cronbach's α, while correlations between variables were established through Pearson's *r* (Table 3). Furthermore, in achieving the research objectives, the study conducted a comprehensive analysis of the proposed research model using structural equation modeling (SEM)

**Table 1:** Differences of performance in hospital nurses according to subject's general characteristics (*N* = 314).

Characteristics	*n* (%)/mean(SD)	Nursing performance		
		Mean(SD)	*t*(*F*)	*P*
Age (years)	31.6(7.4)			
20–29	161(51.3)	3.64(0.46)a	10.61	<0.001
30–39	91(29.0)	3.75(0.56)b	a,b<c	
40–59	62(19.7)	3.99(0.51)c		
Clinical experience (months)	94.4(87.3)			
1–12	42(13.4)	3.50(0.57)a	6.58	<0.001
13–36	65(20.7)	3.64(0.45)b	a<d	
37–60	52(16.6)	3.73(0.49)c		
61–	155(49.4)	3.85(0.50)d		
Gender				
Male	15(4.8)	3.59(0.32)	1.18	0.237
Female	299(95.2)	3.75(0.52)		
Education				
College	36(11.5)	3.52(0.36)a	5.64 a<b,c	0.004^⁎⁎^
University	232(73.8)	3.74(0.51)b		
Master & Dr. degree	46(14.7)	3.94(0.60)c		
Marital status				
No	208(66.2)	3.68(0.50)a	3.05	0.002^⁎⁎^
Yes	106(33.8)	3.87(0.53)b		
Type of Hospital				
Tertiary	134(42.7)	3.76(0.53)	0.43	0.664
General	180(57.3)	3.73(0.51)		
Position				
Nurse	255(81.2)	3.71(0.52)	2.99	0.052
Charge	33(10.5)	3.86(0.47)		
Head	26(8.3)	3.92(0.45)		
Department				
General ward	190(60.5)	3.71(0.49)	0.82	0.530
ICU	26(8.3)	3.76(0.54)		
ER	6(1.9)	3.67(0.50)		
OR	10(3.2)	3.71(0.29)		
OPD	48(15.3)	3.87(0.63)		
Others	34(10.8)	3.77(0.51)		
Working type				
shift	223(71.0)	3.67(0.47)a	4.11	<0.001
Regular	91(29.0)	3.93(0.58)c		
Nationally designated hospital beds				
Yes	287(91.4)	3.76(0.52)	1.70	0.089
No	27(8.6)	3.58(0.45)		
Education on emerging infectious diseases				
Yes	213(67.8)	3.79(0.49)	1.96	0.052
No	101(32.2)	3.65(0.55)		
Education on PPE				
Yes	258(82.2)	3.77(0.49)	1.49	0.139
No	56(17.8)	3.63(0.62)		
Nursing experience with confirmed COVID-19				
Yes in 1 month	144(45.9)	3.76(0.47)	0.39	0.676
Yes 1 more month ago	61(19.4)	3.76(0.56)		
No	109(34.7)	3.71(0.55)		
Total	314(100)	3.74(0.52)		

*Abbreviation*: ICU (Intensive Care Unit), ER (Emergency Room), OR (Operating Room), OPD (Outpatient Department), Standard deviation(SD), Personal protective equipment (PPE)

Calculated by *t*-test, ANOVA and Sheffe test.

*P < 0.05, ^⁎⁎^*P* < 0.01.

### 2.5 Research model

**Fig. 1:**
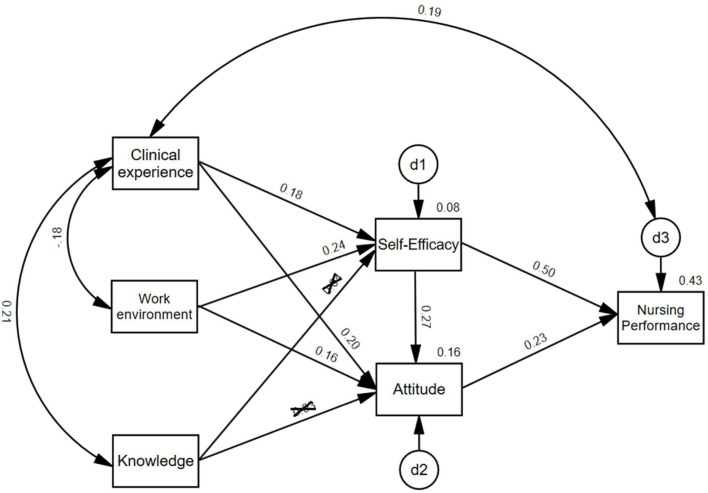
Research model. Two-way arrows are correlations, and one-way arrows are regression relationships. X marks on numbers mean statistical insignificance. “Clinical experience” = Total length of clinical career, “Knowledge” = Knowledge of COVID-19, “Attitude” = Attitude toward COVID-19. Model Fit was excellent: *x*^2^ = 3.15 (*p* > 0.05), *df* = 3, CMIN/DF = 1.05, RMR = 11.03, GFI = 0.99, AGFI = 0.98, NFI = 0.99, CFI = 0.99, TLI = 0.99, RMSEA = 0.01 (HI = 0.10, LO = 0.00). d1–d3 are exogenous variables that the study did not measure but were statistically estimated.

Utilizing SEM, the model depicted in Fig. 1 was analyzed to assess the factors influencing nursing performance during COVID-19. Nursing performance, the primary dependent variable, is directly influenced by self-efficacy and attitudes towards COVID-19, consistent with previous studies. Clinical experience, work environment, and knowledge about COVID-19 are indirect predictors, affecting performance through self-efficacy and attitude. This reflects the complex dynamics noted in nursing research conducted during the pandemic. For example, Pragholapati (2020) and Lim et al. (2022) highlighted self-efficacy's impact on nursing performance. Yang et al. (2022) identified clinical experience's indirect effects through self-efficacy and professional attitude. Lim et al. (2022) discussed how knowledge and work environment indirectly affect performance via self-efficacy and professional attitude. Alshammari and Alenezi (2023) explored how clinical experience and work environment impact performance through self-efficacy and social support. This modeling approach comprehensively assesses both direct and mediated effects, offering robust analysis of factors enhancing or impeding nursing efficacy in crises.

The hypothetical relationships are theoretically supported by Social Cognitive Theory (SCT) (Bandura, 1986). SEM aligns with SCT, emphasizing self-efficacy's role in nursing performance during COVID-19. SCT posits that behavior results from the interplay between personal factors, environmental contexts, and the behavior itself. The research model positions self-efficacy and attitudes as direct influences on performance, reflecting SCT's focus on self-beliefs under stress. Indirect predictors like clinical experience, work environment, and knowledge illustrate how these elements shape self-efficacy and attitudes, impacting performance through mediation. This approach highlights the direct impact of self-efficacy on performance, as shown by Pragholapati (2020) and Lim et al. (2022), and the mediated influence of environmental and experiential factors, as indicated by Yang et al. (2022), Lim et al. (2022), and Alshammari and Alenezi (2023).

SEM's use in exploring nursing performance factors during COVID-19 is due to its effectiveness in evaluating complex relationships between variables. SEM facilitates the investigation of both direct and indirect effects, providing a detailed understanding of how self-efficacy, work environment, knowledge, and attitudes towards COVID-19 affect nursing performance. This method's ability to assess multiple dependent relationships simultaneously reveals the collective impact of these factors on nursing outcomes during health emergencies.

Moreover, SEM accounts for compounding effects and mediating factors based on the structure of the research model, as depicted in Fig. 1 and Table 5. It delineates the results into direct, indirect, and total effects. Furthermore, instead of presenting confidence intervals, structural equation modeling provides critical ratios for each direct effect (i.e., regression weight) in addition to *p*-values, as illustrated in Table 4. A common threshold used for significance testing is a critical ratio value of ±1.96 (*z*-score), which corresponds to a significance level of 0.05 (Witte and Witte, 2017). If the absolute value of the critical ratio is greater than 1.96, it suggests that the parameter estimate is statistically significant at the 0.05 level.

For model fit evaluation, absolute fit indices such as x², normed *x*², RMR, RMSEA, GFI, and AGFI were examined. Additionally, incremental fit indices like NFI, CFI, and TLI were analyzed. A smaller or non-significant *x*² suggests better model conformity. Indices like GFI, AGFI, NFI, CFI, and TLI achieving values of 0.90 or higher indicate satisfactory model fit.

Upon scrutinizing the research model (Fig. 1), attention was given to aspects like regression weights/factor loadings, standard errors, critical ratios, *p*-values, and squared multiple correlations (*R*²). The study then delved into direct, indirect, and cumulative effects across the variables. All data analyses were executed using the SPSS 26 and AMOS 28 software packages.

## 3 Results

### 3.1 General characteristics

#### 3.1.1 Characteristics

Table 1 elucidates the general characteristics of the study participants. The mean age of participants was 31.6 (standard deviation = 7.4) years. The age distribution revealed that 51.3 % were in their 20 s, 29.0 % in their 30 s, and 19.7 % were aged 40 or older. The average duration of clinical experience stood at 94.4 months, segmented as follows: 13.4 % had 12 months or less, 20.7 % ranged between 13–36 months, 16.6 % between 37–60 months, and 49.4 % had more than 60 months.

Gender distribution showed a higher representation of females at 95.2 %, while males constituted 4.8 %. In terms of educational background, 73.8 % held a university degree, 14.7 % had obtained a master's degree or higher, and 11.5 % had a community college degree. Reflecting on marital status, singles represented 66.2 % and the married cohort was 33.8 %.

Workplace characteristics were diverse. 57.6 % of the participants worked in general hospitals, with the remainder, 42.4 %, in tertiary hospitals. The majority, at 81.2 %, held positions as general nurses. Work shifts were divided between 71 % of the participants working in shifts, while 29.0 % adhered to regular working hours.

Training and hands-on experience in the context of the ongoing pandemic were also assessed. A significant 91.4 % were affiliated with hospitals that have nationally designated isolation beds. Two-thirds, or 67.8 %, had received education on emerging infectious diseases, and a larger fraction, 82.2 %, had been trained on the usage of personal protective equipment. In the context of direct exposure, 45.9 % had experience caring for confirmed COVID-19 cases within the last month, 19.4 % had such experience but it dated back to more than a month ago, and 34.7 % had not cared for any confirmed COVID-19 cases.

#### 3.1.2 Performance according to the characteristics

Nursing performance varied based on factors such as age, clinical experience, education, marital status, and work shift types, as illustrated in Table 1. Participants aged 40 or older had a mean nursing performance score of 3.99, which stood out as significantly higher than the scores of those aged 20–29 (3.64) and 30–39 (3.75) years (*f* = 10.67, *p <* 0.001). When considering clinical experience, those with over 61 months showcased a performance score of 3.85, notably surpassing those with 12 months or less at 3.50 (*f* = 6.58, *p <* 0.001). From an educational perspective, individuals holding a master's degree or higher had a score of 3.90, significantly above those with community college degrees, who scored 3.52 (*f* = 5.64, *p* = 0.004). In terms of marital status, married nurses recorded a performance score of 3.87, higher than the 3.68 score of single nurses (*t* = 3.05, *p* = 0.002). Regarding work shifts, daytime workers achieved a performance score of 3.93, markedly better than those on shift work, who scored 3.67 (*t* = 4.11, *p <* 0.001). However, the presence or absence of education on new infectious diseases did not demonstrate a statistically significant difference in nursing performance.

#### 3.1.3 Average scores of the variables

The findings, detailed comprehensively in Table 2, present both total scores and Likert score means for each variable to enhance methodological clarity and provide more precise understanding. The total score for self-efficacy averages 64.5 (SD = 8.6) out of a maximum of 85, indicating a generally competent level among participants, with a corresponding Likert score mean of 3.79 (SD = 0.50) out of 5.0, suggesting a score above the median. This affirms a robust sense of self-efficacy. The work environment is reflected by a total score mean of 71.7 (SD = 12.9) out of a maximum of 116, indicating average quality, and a Likert score mean of 2.47 (SD = 0.44) out of 4.0, positioning it near the scale's midpoint. Knowledge about COVID-19 is shown by a total score mean of 13.4 (SD = 2.5) out of a possible 20, with the Likert score mean of 0.67 (SD = 0.12) out of a maximum of 1.0 elucidating the proportional correctness per question, highlighting areas of strength and improvement. The attitude toward COVID-19 is particularly positive, with a total score mean of 48.7 (SD = 4.8) out of 60 and a Likert score mean of 4.06 (SD = 0.40) out of 5, underscoring an effective approach to prevention and control. Lastly, nursing performance is indicated by a total score mean of 168.6 (SD = 23.4) out of 225 and a Likert score mean of 3.74 (SD = 0.52) out of 5.0, suggesting that participants are reasonably proficient in performing necessary tasks, with performance levels above the median.

**Table 2:** Subjects’ self-efficacy, work environment, knowledge, attitude, and nursing performance (*N* = 314).

Variables	Total score		Likert score	
	mean (SD)	range	mean (SD)	range
Self-efficacy	64.5(8.6)	26–85	3.79(0.50)	1.53–5
Work environment	71.7(12.9)	38–113	2.47(0.44)	1.31–3.9
Knowledge	13.4(2.5)	7–19	.67(0.12)	.35–0.95
Attitude	48.7(4.8)	18–60	4.06(0.40)	1.50–5
Nursing performance	168.6(23.4)	74–225	3.74(0.52)	1.64–5

*Note*. Knowledge = Knowledge of COVID-19, Attitude = Attitude toward COVID-19.

Values are presented as mean (SD) score.

These dual presentations of total and Likert scores are chosen to cater to specific applications of each scoring method in the analysis. Total scores are utilized directly in structural equation modeling to maintain consistency with other cumulative variables, providing a robust basis for analyzing relationships and effects. The Likert scores offer additional context, enriching the understanding of the distribution of responses and the relative difficulty or ease across different items and domains.

This structured approach to data presentation aims to enhance the transparency of analytical choices and provide a more accessible interpretation of the results.

### 3.2 Correlations of the variables

As illustrated in Table 3, among the variables, self-efficacy exhibited the most robust correlation with nursing performance (*r* = 0.61, *p <* 0.001), closely followed by the attitude related to COVID-19 (*r* = 0.44, *p <* 0.001). Clinical experience also displayed a moderate association with nursing performance (*r* = 0.28, *p <* 0.001). Meanwhile, the nursing work environment (*r* = 0.17, *p* = 0.003) and knowledge related to COVID-19 (*r* = 0.14, *p* = 0.017) demonstrated weaker correlations with nursing performance.

**Table 3:** Correlation between self-efficacy, work environment, knowledge, attitude, and nursing performance (*N* = 314).

Variables	Clinical experience	Self -efficacy	Work environment	Knowledge	Attitude	Nursing performance
Clinical experience	1					
Self-efficacy	0.146*	1				
Work environment	−0.176^⁎⁎^	0.208^⁎⁎^	1			
Knowledge	0.230^⁎⁎^	0.080	0.009	1		
Attitude	0.213^⁎⁎^	0.331^⁎⁎^	0.180^⁎⁎^	0.096	1	
Nursing performance	0.279^⁎⁎^	0.607^⁎⁎^	0.166^⁎⁎^	0.135*	0.435^⁎⁎^	1

*Note*. Clinical experience = Total length of clinical career, Knowledge = Knowledge of COVID-19, Attitude = Attitude toward COVID-19.

Calculated by Pearson's correlation coefficient.

P* < 0.05, p** < 0.01, p*** < 0.001.

### 3.3 Research model

#### 3.3.1 Research model structure

Utilizing structural equation modeling, the research model depicted in Fig. 1 was explored. In this model, nursing performance serves as the primary dependent variable, while the total length of clinical experience, knowledge of COVID-19, attitude toward COVID-19, self-efficacy, and work environment act as independent variables. Specifically, due to their strong correlation with nursing performance as shown in Table 3, self-efficacy and attitude function as direct predictors. Conversely, variables demonstrating weaker or moderate associations with nursing performance, namely clinical experience, work environment, and knowledge (refer to Table 3), serve as indirect predictors to nursing performance.

#### 3.3.2 Model fit & results

The findings are detailed in Fig. 1, Tables 4, and 5. As depicted in Fig. 1, the model exhibits an excellent fit with indices such as *x*^2^ = 3.15 (*p* > 0.05), *df* = 3, CMIN/DF = 1.05, RMR = 11.03, GFI = 0.99, AGFI = 0.98, NFI = 0.99, CFI = 0.99, TLI = 0.99, and RMSEA = 0.01 (HI = 0.10, LO = 0.00).

**Table 4:** Standardized regression weights (*N* = 314).

Dependent variables	Independent variables	Standardized factor loading values	SE	CR	*p-*value
Self-efficacy	Clinical experience	0.179**	0.006	3.160	0.002^⁎⁎^
	Work environment	0.239***	0.037	4.328	<0.001^⁎⁎^
	Knowledge	insignificant	0.194	0.660	0.509
Attitude	Clinical experience	0.195***	0.003	3.557	<0.001^⁎⁎^
	Work environment	0.159**	0.020	2.926	0.003^⁎⁎^
	Knowledge	insignificant	0.104	0.527	0.598
	Self-efficacy	0.267***	0.030	4.949	<0.001^⁎⁎^
Nursing performance	Self-efficacy	0.504***	0.122	11.291	<0.001^⁎⁎^
	Attitude	0.232***	0.219	5.133	<0.001^⁎⁎^

*Note*. Estimate = Standardized Regression weights, SE = Standard Error, CR = Critical Ratio.

Clinical experience = Total length of clinical career, Knowledge = Knowledge of COVID-19, Attitude = Attitude toward COVID-19; p* < 0.05, p** < 0.01, p*** < 0.001. Calculated by SEM.

**Table 5:** Direct, indirect, and total effects between variables (*N* = 314).

Dependent variables	Independent variables	Direct effects (standardized) *β*_direct_	Indirect effects (standardized) *β*_indirect_	Total effects (standardized) *β*_total_
Self-efficacy	Clinical experience	0.179**	n/a	0.179
	Work environment	0.239***	n/a	0.239
	Knowledge	insignificant	n/a	n/a
Attitude	Clinical experience	0.195***	0.048	0.243
	Work environment	0.159**	0.064	0.223
	Knowledge	insignificant	0.010	0.010
	Self-efficacy	0.267***	n/a	0.267
Nursing performance	Self-efficacy	0.504***	0.062	0.566
	Attitude	0.232***	n/a	0.232
	Knowledge	n/a	0.027	0.027
	Work environment	n/a	0.172	0.172
	Clinical experience	n/a	0.147	0.147

*Note*. Clinical experience = Total length of clinical career, Knowledge = Knowledge of COVID-19, Attitude = Attitude toward COVID-19; p* < 0.05, p** < 0.01, p*** < 0.001. Calculated by SEM.

Two key predictors of nursing performance are attitude and self-efficacy, accounting for 43 % (*R*^2^ = 0.43) of the variance as indicated in Fig. 1. Conversely, work environment (*β*_indirect_ = 0.17) and clinical experience (*β*_indirect_ = 0.15) exhibit limited indirect effects on nursing performance (Table 5). Knowledge does not statistically significantly predict nursing performance (no *β*_direct_ or *β*_indirect_) (Table 5).

As shown in Fig. 1 and Table 4, self-efficacy was positively predicted by clinical experience (*β*_direct_ = 0.18, CR = 3.16, *p* = 0.002) and work environment (*β*_direct_ = 0.24, CR = 4.33, *p <* 0.001), while attitude was similarly influenced (*β*_direct_ = 0.20 and 0.16, CR = 3.56 and 2.93, respectively). However, clinical experience and work environment only explained 8 % (*R*^2^ = 0.08) of self-efficacy and 16 % (*R*^2^ = 0.16) of attitude. Knowledge about COVID-19 exhibited a statistically insignificant influence on self-efficacy (CR = 0.66, *p* = 0.509) or attitude (CR = 0.53, *p* = 0.60).

Table 4, Table 5 elucidate the standardized values of all effects in the research model. Direct effects on nursing performance are observed from self-efficacy (*β*_direct_ = 0.50, CR = 11.30, *p <* 0.001) and attitude (*β*_direct_ = 0.23, CR = 5.13, *p <* 0.001), which jointly explain 43 % (*R*^2^) of the variance as described above. Self-efficacy also exerts an indirect influence on nursing performance mediated by attitude, with a value of *β*_indirect_ = 0.06. Indirect effects on nursing performance are seen from clinical experience (*β*_indirect_ = 0.15) and work environment (*β*_indirect_ = 0.17).

In terms of self-efficacy, direct effects come from clinical experience (*β*_direct_ = 0.18) and work environment (*β*_direct_ = 0.24), together accounting for 8 % of the variance (*R*^2^). Similarly, clinical experience (*β*_direct_ = 0.20) and work environment (*β*_direct_ = 0.16) directly influence attitude, explaining 16 % (*R*^2^) of the variance. Indirect effects on attitude are noted from clinical experience (*β*_indirect_ = 0.05) and work environment (*β*_indirect_ = 0.06). Contrarily, knowledge does not impact any of the aforementioned variables. To provide a comprehensive view, total effects were computed by amalgamating associated direct and indirect effects, as detailed in Table 5.

## 4 Discussion

The study examined factors affecting nursing performance among 314 Korean nurses during COVID-19. Performance varied by age, clinical experience, education, marital status, and work shifts (Table 1). Clinical practice experience, self-efficacy, work environment, and attitudes toward COVID-19 correlated with performance (Table 3). Self-efficacy and attitudes were significant predictors, explaining 43 % of the variance (Fig. 1). Indirect effects on performance were mediated by self-efficacy and attitude, while COVID-19 knowledge was not statistically significant (Table 4).

### 4.1 General characteristics

Nursing performance among nurses varied based on factors such as age, clinical experience, education, marital status, and work shifts (Table 1). Performance was found to be superior among nurses aged 40 or older compared to their younger counterparts (*f* = 10.61, *p <* 0.001), those with over 61 months of clinical experience versus 12 months or less, and those possessing master's degrees as opposed to community college degrees (*f* = 6.58, *p <* 0.001). Married nurses outperformed their non-married peers (*t* = 3.05, *p* = 0.002), and daytime workers excelled over shift workers (*t* = 4.11, *p <* 0.001). These findings align with prior studies (Jo and Sung, 2018; Song et al., 2006). For instance, Jo and Sung (2018) observed significant performance variations among 140 nurses based on age, clinical experience, and marital status. Similarly, Song et al. (2006) identified age, clinical experience, education, and marital status as differentiators in nursing performance among 830 nurses. Lim et al. (2022) found that nurses who were older, married, and had higher levels of education exhibited significantly better job performance during the COVID-19 pandemic. Further supporting the importance of clinical experience and education, Park et al. (2021) and Duran et al. (2021) found that crisis response abilities were enhanced among nurses with prolonged experience and advanced education, highlighting their importance during infectious disease outbreaks.

Contrastingly, the influence of training on nursing performance in this study diverged from previous findings. While Park and Kim (2011) reported that nurses trained in job manuals displayed notably higher performance, the current study found no statistically significant performance differences among nurses based on training concerning emerging infectious diseases or personal protective equipment. This disparity may be attributed to the study's timing—approximately a year post-pandemic onset—and the frequent alterations in guidelines due to evolving information on COVID-19. Moreover, the pervasive use of personal protective equipment from the pandemic's onset, irrespective of formal training, might have leveled the performance differences based on such education.

### 4.2 Primary variables

A significant feature of this study is its contextualization within the backdrop of the COVID-19 pandemic, and its findings hold the potential to be instrumental in the preparation of nurses for future emerging infectious diseases. Self-efficacy (*β*_direct_ = 0.50, CR = 11.30, *p <* 0.001) and attitudes toward COVID-19 emerge (*β*_direct_ = 0.23, CR = 5.13, *p <* 0.001) as the most salient determinants of nursing performance in this study with robust explanatory power of *R*^2^ = 43 % (*R*^2^) (Tables 4, 5, and Fig. 1). This is consistent with Jo and Sung's (2018) findings, where self-efficacy was identified as a primary driver of nursing performance among emergency room nurses. The pronounced explanatory power of self-efficacy and attitudes over knowledge underscores the pivotal role of a positive mindset and robust self-belief in effective nursing performance, especially in the context of new infectious diseases. Hence, this study underscores the imperative for nurses to possess elevated self-efficacy and maintain a constructive attitude when addressing novel infectious diseases.

The findings of this study strongly align with Social Cognitive Theory (SCT), emphasizing the significant roles of self-efficacy and attitudes towards COVID-19 in influencing nursing performance. Consistent with SCT, which suggests that behavior is influenced by personal beliefs, behaviors, and environmental contexts, the current study's results highlight self-efficacy (*β*_direct_ = 0.50, CR = 11.30, *p <* 0.001) and attitudes towards the pandemic (*β*_direct_ = 0.23, CR = 5.13, *p <* 0.001) as potent determinants of effective nursing. This supports SCT's view that personal cognitions shape behavioral outcomes, particularly in high-stress environments like those presented by the COVID-19 pandemic. These insights suggest that enhancing self-efficacy and positive attitudes should be central in preparing nurses for future health crises, reflecting SCT's focus on the importance of psychosocial factors over mere knowledge accumulation.

In the context of the COVID-19 pandemic, the present study identified both self-efficacy and attitudes towards COVID-19 as critical factors influencing nursing performance. Previous research highlights that self-efficacy enables nurses to effectively manage stress and anxiety under pandemic pressures, enhancing resilience and overall performance. For example, Pragholapati (2020) emphasizes that high self-efficacy equips nurses to navigate the adversities of high-stress environments, while Lim et al. (2022) discuss the moderating effect of burnout, suggesting that maintaining self-efficacy is essential even when facing occupational burnout. These findings advocate for the integration of strategies in nursing training that enhance self-efficacy and cultivate positive attitudes, thereby fostering a workforce capable of delivering effective care in challenging healthcare crises. Similarly, attitudes towards COVID-19 significantly correlate with performance, where a positive mindset directly enhances care quality. Yulianti (2021) further emphasized that positive attitudes not only improve job performance but also maintain job satisfaction among nurses, particularly in high-pressure pandemic conditions. Collectively, these insights underscore the necessity of embedding positive psychological attributes into nurse training programs to sustain high performance during health crises (Pragholapati, 2020; Lim et al., 2022; Yulianti, 2021).

Despite the marginal effects of clinical experience, work environment, and knowledge on nursing performance, these findings can still be interpreted through the lens of Social Cognitive Theory (SCT), which emphasizes the interaction between personal, behavioral, and environmental factors. While clinical experience and work environment show limited indirect effects on performance (*β*_direct_ = 0.15 and *β*_direct_ = 0.17, respectively), their influence on self-efficacy and attitudes suggests that these environmental factors still play a role, albeit a smaller one than expected, in shaping nursing behavior. This could indicate that during the acute phase of the COVID-19 pandemic, the overwhelming impact of personal factors such as self-efficacy and attitudes may overshadow the typically significant influences of environment and experience. The minimal impact of knowledge about COVID-19 aligns with this interpretation, suggesting that in crisis conditions, practical skills and psychological resilience are more critical than theoretical understanding alone. These observations call for a reevaluation of how environmental factors are integrated into nursing training and practice, especially in emergency and pandemic responses.

The presented study also identified a statistically significant correlation of clinical experience (*r* = 0.28, *p <* 0.001) and self-efficacy (*r* = 0.61, *p <* 0.001) to nursing performance (Table 3). These findings mirror previous research. Yang et al. (2022) highlighted how nursing students' clinical experience indirectly influenced their performance, mediated by self-efficacy and professional attitude, during the COVID-19 pandemic. Alshammari and Alenezi (2023) claimed nurses' clinical experience and work environment affect their performance, with self-efficacy and social support acting as mediating factors. Jo and Sung (2018) found a positive correlation between nurses' self-efficacy in general emergency rooms and their nursing performance, identifying self-efficacy as a pivotal predictor. Similarly, a study by Kim and Lee (2019) on 130 university hospital nurses underscored the positive correlation between self-efficacy and nursing performance. Collectively, both the present and earlier studies underscore the paramount importance of self-efficacy in determining nurses' performance outcomes.

In the current study, knowledge about COVID-19 exhibited a marginal correlation with nursing performance (*r* = 0.14, *p <* 0.05, Table 5). Analysis of the research model (Fig. 1) further indicates that such knowledge is not a statistically significant predictor of other relevant variables, rendering it insufficient to elucidate nursing performance. The limited influence of knowledge might be attributed to the nascent and rapidly evolving nature of medical information during the pandemic's onset (Buheji and Buhaid, 2020). In contrast, Lim et al. (2022) identified that nurses' COVID-19 knowledge indirectly influenced nursing performance, with self-efficacy and burnout associated with professional attitudes during the pandemic. Similarly, Khandan et al. (2024) observed positive correlations between knowledge and performance. The key distinction of the current study lies in its comprehensive approach, as it uniquely integrates clinical experience, knowledge, and work environment into a single model, accounting for their interactive effects, which may explain the differences from previous findings. Conversely, the attitude toward COVID-19 demonstrated a meaningful correlation (*r* = 0.44, *p <* 0.001, Table 3) with nursing performance and emerged as a prominent predictor within the research model (Table 4 and Fig. 1).

This study, set against the backdrop of the COVID-19 pandemic, highlights critical elements for nursing preparedness in the face of emerging infectious diseases. The findings reveal that self-efficacy and attitudes towards COVID-19 are significant determinants of nursing performance, overshadowing the role of knowledge. Incorporating the study's findings into nursing education and training programs necessitates a strategic approach focused on enhancing self-efficacy and cultivating positive attitudes towards infectious disease management. Practical recommendations for educational curricula include implementing simulation-based training to bolster confidence through realistic scenarios, integrating interprofessional education to foster a collaborative and positive work culture, and offering mental health and resilience workshops to equip nurses with stress management techniques. Moreover, embracing technology-enhanced learning tools, such as virtual reality simulations and online platforms, can provide flexible and immersive learning experiences. Continuous professional development opportunities, including webinars and workshops on emerging infectious diseases, should be promoted to encourage lifelong learning. Finally, reflective practice and feedback mechanisms are essential for developing self-awareness and reinforcing the practical application of knowledge. By embedding these elements into nursing education, programs can prepare nurses not only with the necessary clinical skills but also with the psychological resilience and positive mindset crucial for effective healthcare delivery in the face of future infectious disease outbreaks.

For researchers, this underscores the importance of further exploring the dynamics between mindset, beliefs, and performance, especially in the context of new infectious diseases. Additionally, the marginal correlation of COVID-19 knowledge with nursing performance suggests the need for more research into how evolving medical information impacts healthcare delivery during pandemics. These insights are invaluable for refining educational curricula and professional development strategies in nursing, ensuring that future healthcare workers are not only well-informed but also equipped with the resilience and positive mindset necessary to excel in challenging medical situations.

In addition, there was a possibility that external factors such as changing public health guidelines, variable access to resources like personal protective equipment (PPE), and the overall healthcare response to the COVID-19 pandemic have impacted nursing performance (Chemali et al., 2022; Shreffler et al., 2020). These challenges could affect nurses' self-efficacy and attitudes, highlighting the need for systemic support alongside individual training. Recognizing these influences is crucial for understanding nursing performance in pandemic conditions and preparing for future health emergencies.

## 5 Limitations

The study employed a survey method dependent on participants' recall, introducing the potential for discrepancies between actual events and their recollections. While the exclusive focus on Korean nurses aligns with the geographical considerations of COVID-19, it might limit the study's generalizability as the participants were recruited through a social network bulletin in Korea. Additionally, data was gathered through both online and offline methods, which might differentially impact the participants' immediate recall capacities.

## 6 Conclusions

This study provides valuable insights into the factors influencing nursing performance during the COVID-19 pandemic, with a focus on Korean nurses. The research demonstrates that self-efficacy and attitudes toward COVID-19 are the most significant predictors of nursing performance, collectively explaining 43 % of the variance. These findings underscore the importance of psychological resilience and a positive mindset in enhancing nursing outcomes, particularly in the context of emerging infectious diseases.

The study reveals that while clinical experience and work environment have indirect effects on performance, their influence is mediated by self-efficacy and attitudes. Knowledge about COVID-19, while essential, did not significantly predict nursing performance, suggesting that practical skills and psychological factors are more critical during emerging health crises. This highlights the need for nursing education programs to prioritize the development of self-efficacy and positive attitudes, alongside clinical knowledge.

The study confirms that self-efficacy and attitudes are central to nursing performance, particularly under the pressures of a pandemic. These findings align with Social Cognitive Theory, emphasizing the role of personal beliefs and attitudes in shaping behavior and performance in high-stress environments.

The strength of this study lies in its comprehensive approach, integrating both personal and environmental factors into a unified model. This approach provides a nuanced understanding of how these factors interact to influence nursing performance, offering valuable guidance for future nursing education and training programs. By focusing on nurses’ self-efficacy, attitude, and clinical preparedness, healthcare systems can better equip nurses to handle future outbreaks of infectious diseases.

## Funding

This work was supported by the National Research Foundation of Korea (NRF) grant funded by the Korea government (MSIT) (No. 2020R1F1A1055437).

## CRediT authorship contribution statement

**Kyung-sook Cha:** Writing – review & editing, Writing – original draft, Supervision, Resources, Project administration, Methodology, Investigation, Funding acquisition, Data curation, Conceptualization. **Dohyun Lee:** Writing – review & editing, Writing – original draft, Visualization, Validation, Software, Methodology, Investigation, Data curation.

## Declaration of competing interest

The authors declare that they have no conflict of interest.

## Data Availability

The authors can provide the data at the journal editor's or reviewer's request.
